# On-Growth and In-Growth Osseointegration Enhancement in PM Porous Ti-Scaffolds by Two Different Bioactivation Strategies: Alkali Thermochemical Treatment and RGD Peptide Coating

**DOI:** 10.3390/ijms23031750

**Published:** 2022-02-03

**Authors:** Katrin Steffanie Rappe, Monica Ortiz-Hernandez, Miquel Punset, Meritxell Molmeneu, Albert Barba, Carles Mas-Moruno, Jordi Guillem-Marti, Cristina Caparrós, Elisa Rupérez, José Calero, María-Cristina Manzanares, Javier Gil, Jordi Franch

**Affiliations:** 1 Surgery Department, Veterinary School of Barcelona, Universitat Autònoma de Barcelona, Bellaterra, 08193 Barcelona, Spain; Katrin.Rappe@uab.cat (K.S.R.); albert.barba@uab.cat (A.B.); jordi.franch@uab.cat (J.F.); 2 Biomaterials, Biomechanics and Tissue Engineering Group (BBT), Department of Materials Science and Metallurgical Engineering, Universitat Politècnica de Catalunya (UPC), 08019 Barcelona, Spain; monica.ortiz-hernandez@upc.edu (M.O.-H.); miquel.punset@upc.edu (M.P.); meritxell.molmeneu@upc.edu (M.M.); carles.mas.moruno@upc.edu (C.M.-M.); jordi.guillem.marti@upc.edu (J.G.-M.); cristina.caparros@upc.edu (C.C.); elisa.ruperez@upc.edu (E.R.); 3 Barcelona Research Center in Multiscale Science and Engineering, Universitat Politècnica de Catalunya (UPC), 08019 Barcelona, Spain; 4 Research Centre for Biomedical Engineering (CREB), Universitat Politècnica de Catalunya, 08034 Barcelona, Spain; 5 Institut de Recerca Sant Joan de Déu (IRSJD), 08034 Barcelona, Spain; 6 UPC Innovation and Technology Center (CIT-UPC), Technical University of Catalonia (UPC), C. Jordi Girona 3-1, 08034 Barcelona, Spain; 7 AMES GROUP S.A., Carretera Nacional 340, Pol. Ind. “Les Fallulles”, 08620 Sant Vicenç dels Horts, Barcelona, Spain; jacalero@ames.es; 8 Human Anatomy and Embryology Unit, Department of Pathology and Experimental Therapeutics, Universitat de Barcelona, L’Hospitalet de Llobregat, 08907 Barcelona, Spain; mcmanzanares@ub.edu; 9 Facultad de Odontología, Campus de Medicina y Ciencias de la Salud, Universidad Internacional de Cataluña (UIC), 08017 Barcelona, Spain; 10 Bioengineering Institute of Technology, Universitat Internacional de Catalunya, 08195 Sant Cugat del Vallés, Barcelona, Spain

**Keywords:** titanium foams, osseointegration, thermochemical treatment, RGD peptide, in vivo implantation, histomorphometric evaluation, bone on-growth, bone in-growth

## Abstract

A lack of primary stability and osteointegration in metallic implants may result in implant loosening and failure. Adding porosity to metallic implants reduces the stress shielding effect and improves implant performance, allowing the surrounding bone tissue to grow into the scaffold. However, a bioactive surface is needed to stimulate implant osteointegration and improve mechanical stability. In this study, porous titanium implants were produced via powder sintering to create different porous diameters and open interconnectivity. Two strategies were used to generate a bioactive surface on the metallic foams: (1) an inorganic alkali thermochemical treatment, (2) grafting a cell adhesive tripeptide (RGD). RGD peptides exhibit an affinity for integrins expressed by osteoblasts, and have been reported to improve osteoblast adhesion, whereas the thermochemical treatment is known to improve titanium implant osseointegration upon implantation. Bioactivated scaffolds and control samples were implanted into the tibiae of rabbits to analyze the effect of these two strategies in vivo regarding bone tissue regeneration through interconnected porosity. Histomorphometric evaluation was performed at 4 and 12 weeks after implantation. Bone-to-implant contact (BIC) and bone in-growth and on-growth were evaluated in different regions of interest (ROIs) inside and outside the implant. The results of this study show that after a long-term postoperative period, the RGD-coated samples presented higher quantification values of quantified newly formed bone tissue in the implant’s outer area. However, the total analyzed bone in-growth was observed to be slightly greater in the scaffolds treated with alkali thermochemical treatment. These results suggest that both strategies contribute to enhancing porous metallic implant stability and osteointegration, and a combination of both strategies might be worth pursuing.

## 1. Introduction

The objective of a wide range of medical and veterinary disciplines for many centuries has been to provide implants with proper functioning and an adequate biological response. Suitable biomechanical properties are sought for bone application biomaterials to stimulate bone tissue growth and consolidation.

According to previous studies, various properties of bone-mimicking biomaterials need to be simultaneously adjusted for optimal bone tissue regeneration and implant osseointegration [1,2]. Cell activity and bone tissue formation are stimulated by several factors, such as physical substrate topography, geometrical design, shear stress, stiffness, and electrical forces, as well as biochemical elements, such as growth factors, genes, and proteins [3,4,5,6]. Consequently, next-generation tissue-engineered scaffolds must integrate a range of biological and physical properties for optimal bone regeneration. It is important that materials possess optimal biocompatibility, osseoconduction, and osseointegration properties to achieve rapid implant fixation without causing adverse reactions or rejections [7,8,9]. Calcium phosphates, bioglass, and ceramics have been proven to be biocompatible and to have osteoconductive properties through the formation of hydroxyapatite on their surfaces [10]. However, they are biomechanically insufficient as they do not support high stress loads [11,12].

Plain metals, such as titanium, support mechanical loads but do not allow there to be good contact between newly formed bone tissue and the implant surface. Therefore, porous titanium implants with open, interconnected macro- and micropores were developed to allow bone in-growth to penetrate the implant and bone on-growth over the surface of the implant, to improve its mechanical interlocking fixation [13,14,15,16,17,18]. Nowadays, porous titanium and its alloys are commonly used for clinical applications under loading conditions because one can adjust their porosity and mechanical features in order to mimic the bone properties for each site of implantation. This enables its use for orthopedic applications, such as fixation screws, spinal fixation devices, artificial ligament anchors, dental implants, and foot and ankle reconstructive wedges [19,20,21,22].

Three-dimensional open- and interconnected pores promote the penetration of bone-forming cells into the implant, and facilitate proper nutrient supply, due to their large inner volume, for bone in-growth. Subsequently, cell attachment and the proliferation of properly vascularized new bone is achieved, providing a strong and durable implant–bone interaction that enhances the optimal function and the lifetime of the implanted device [17,23,24,25,26].

The scaffold’s geometrical external design and its surface macroroughness provide immediate primary mechanical stability to the implant. The high friction forces featured between the rough implant surface and the peri-implant bone tissue increase the implant’s fixation within its location. Furthermore, the metal structure′s porosity reduces the implant’s Young’s modulus, improving load transfer to adjacent bone tissues. It also reduces the risk of implant loosening through the mitigation and/or reduction of deleterious stress shielding related to bone resorption [22,27,28,29,30,31,32,33,34,35].

Porous titanium scaffolds can be manufactured using a wide variety of processes [6,36], the following being among the most noteworthy: traditional compression and sintering [16,27,37], polymeric sponge replication [38], combustion synthesis [39], powder metallurgy (PM) [16,26,40,41,42,43], rapid prototyping [36,40,44], selective electron beam melting (SEBM) [45,46,47,48,49,50], selective laser melting (SLM) [15,43,51,52,53,54,55,56], and selective laser sintering [36,56,57]. PM seems to be a particularly advantageous method for manufacturing complex shapes with interconnected pores without the need for machining steps [16,42,58], thus shortening the processing route and decreasing related costs [42,58,59]. Pores of PM manufactured scaffolds can be created by different modes, such as particle arrangement during the first compaction step, using spacer particles for disintegration, and by solid-state diffusion during the sintering stage [60]. Moreover, porosity properties, such as pore size, total porosity, pore size distribution, porosity gradient, and porosity interconnectivity can be optimized using this technique in order to better match the bone mechanical properties [16,26,61].

Porous titanium implants alone have been shown to have osseoconductive and osseointegrative properties. However, many treatments are presently available to bioactivate these implants, thus improving their properties for accelerated osseointegration. Ti can also undergo a wide range of surface modifications in order to improve cell adhesion, proliferation, and differentiation, to enhance its osseoconductive and osseointegrative capabilities [6,30,62,63,64,65,66]. In this regard, the surface of the porous system can be activated by different functionalizing coatings using calcium phosphates [67,68,69,70], demineralized bone matrix [26], decellularized bone (DCB) [25], bone marrow aspirate [71], platelet-rich plasma [72], bone morphogenetic proteins [73,74], mesenchymal stem cells [75,76] and bioactive peptides [77,78,79,80,81,82,83].

In this study, highly interconnected porous Ti scaffolds, obtained from PM treated with two different strategies of surface activation, have been evaluated. One of the strategies consists of an inorganic thermochemical treatment that promotes the nucleation and growth of a bone-like apatite layer over the Ti surface [61,84,85,86]. The other strategy involves surface coating by the grafting of a cell adhesive tripeptide (RGD) [26,87,88,89,90]. RGD peptides exhibit an affinity for integrins expressed by osteoblasts, and have been reported to improve osteoblast adhesion and improve implant osseointegration [91,92].

The evaluation of both strategies was performed and compared by means of an in-vivo study, using rabbit tibiae as the animal model. Highly interconnected porous Ti scaffolds obtained from PM without treatment were used as controls. Two time points were studied in order to evaluate the results at short and long implantation times (4 and 12 weeks).

The results of this study showed that after a long-term postoperative period, the peptide-treated samples showed higher values of quantification in the outer area. In the inner area, more variability was observed in the different groups; in terms of total bone in-growth, it was seen that the scaffolds constructed with the thermochemical treatment had quantification values slightly greater than those obtained for the peptide group.

## 2. Results

### 2.1. Clinical and Radiographic Results

All animals recovered uneventfully from the surgery, and no postoperative complications, such as foreign body reaction, infection, etc., were observed. During the study, none of the experimental animals had clinical signs of disease, disturbances of gait, or alterations in their hematologic or biochemical profiles, that could interfere with the study.

**Figure 1:**
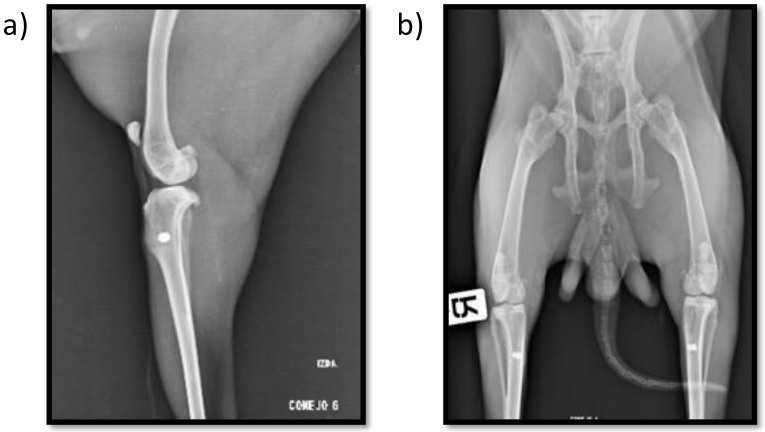
Mediolateral (**a**) and craniocaudal (**b**) postoperative X-ray images showing both insertion point location and implant alignment.

Postoperative radiographs show the proper placement of the titanium implanted cylinders (Figure 1). The radiographic evaluation of the isolated tibiae at the end of the study also showed all the titanium cylinders to be properly placed, with no signs of implant migration.

Peri-implant radiolucency was not observed in any sample, and good bone-to-implant contact was confirmed radiographically in all the samples, indicating an adequate new bone formation response around the implants.

### 2.2. Porous Implant Structure and Mechanical Properties

The porosity of the implants was characterized by mercury immersion porosimetry (MIP), with an interconnected porosity average of 53% formed by macro- and micropores. The mean diameter of the macropores was 210 µm, while micropore size ranged from 1 to 15 µm. The bioactivation process of the scaffolds did not modify the values of titanium porosity in terms of interconnectivity. The characterization of the mechanical properties has been previously described elsewhere [26].

### 2.3. Quantitative Histomorphometric Evaluation

#### 2.3.1. Postoperative Period Groups after 4 Weeks of Implantation

**Figure 2:**
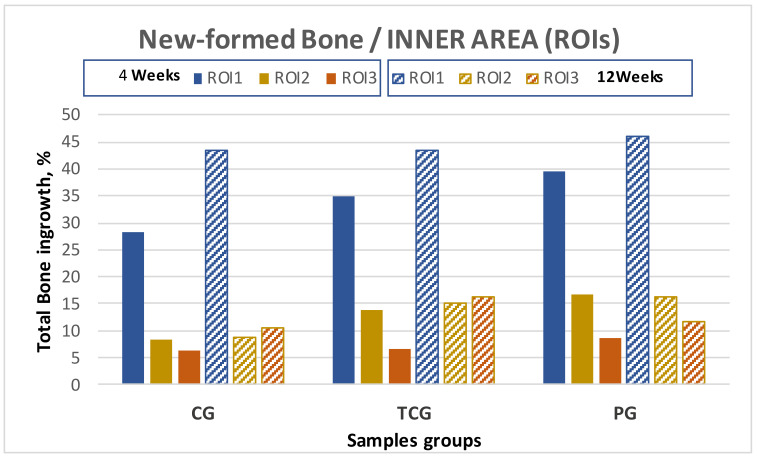
New bone formation in titanium porous foam at 4 and 12 weeks after implantation. There were no statistically significant differences (*p* > 0.05) depending on the type of samples for all analyzed parameters.

The histomorphometry results are presented as the percentage of newly formed bone for every evaluated area, designated as “regions of interest” (ROIs). A total of 15 samples were obtained after 4 weeks of implantation: 5 from the control group, 5 from the thermochemical group, and 5 from the peptide group. All quantitative results for each group are shown in Table 1, together with their standard error of the mean, and graphically presented in (Figure 2).

BSE-SEM observations demonstrated that all implant types showed new bone formation inside the pores after 4 weeks of implantation. No adverse tissue reaction or inflammatory response was observed in the sections examined. The group of implants functionalized with peptides (PAG) obtained the best results for both the outer and inner areas of the implant, showing higher values of bone on-growth (outside the implant), bone in-growth (inside the implant), and BIC than the rest of the groups. Likewise, the thermochemical-treated group (TCG) revealed superior bone formation values when compared to the control group with no superficial treatment (CG), which showed the lowest values for all ROIs. The differences were statistically significant *p* < 0.05.

#### 2.3.2. Postoperative Period Groups 12 Weeks after Implantation

At 12 weeks after implantation, a total of 15 cross-sectional samples were obtained: 5 samples from the control group, 5 samples from the TC, and 5 samples from the peptide group. The results obtained for each group in the different areas are displayed in Table 1.

The samples harvested at the second time point demonstrated a similar trend as those harvested at the first time point, with peptide-treated (PAG) samples exhibiting higher values of bone on-growth in the outer implant area as well as for the BIC.

The results obtained for the evaluated inner ROIs denoted broad variability between the different groups. Thermochemical-treated (TCG) samples showed slightly higher values for the total amount of bone tissues inside the implant than those obtained for the peptide-treated samples. Comparing the different ROIs, the best results for ROI1 and ROI2 were achieved for the peptide (PAG) group, while the thermochemical-treated samples showed the highest results in ROI3.

**Figure 3:**
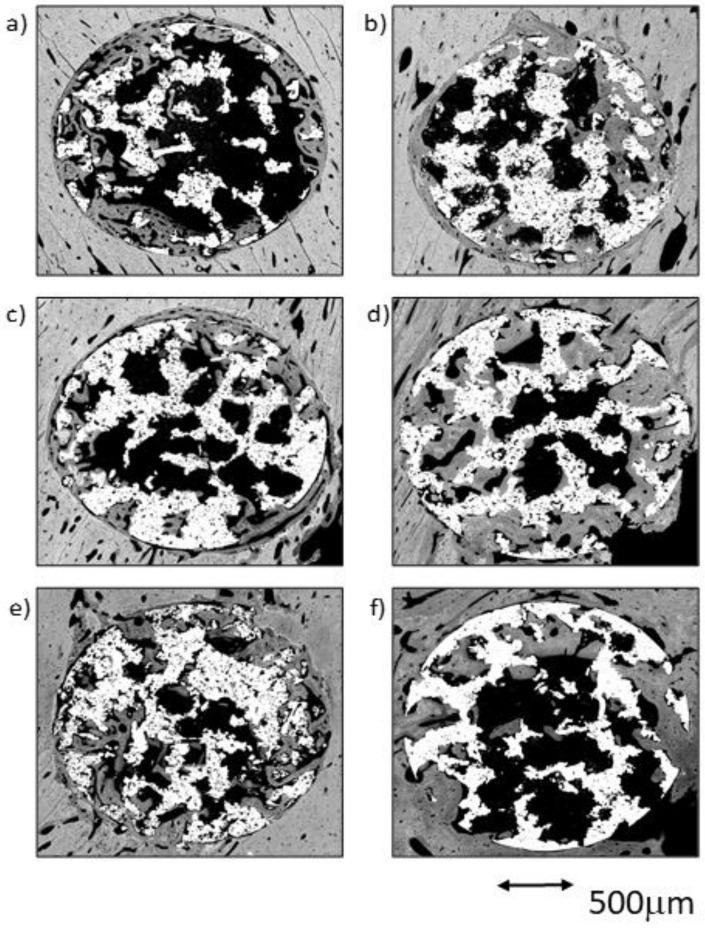
BSE-SEM results 4 weeks (left) and 12 weeks (right) after porous titanium implant insertion in transversal view: (**a**,**b**) CG; (**c**,**d**) TCG; (**e**,**f**) PAG.

**Figure 4:**
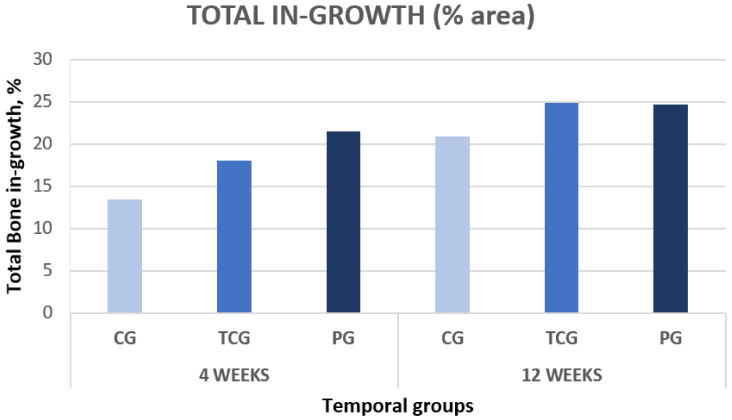
Total new bone formation in titanium porous foam 4 and 12 weeks after implantation.

The comparative analysis between the results for all samples in each group from both time points (4 and 12 weeks) reflected an overall tendency for increases in the newly formed bone percentage during the postoperative period. However, an isolated anomaly was observed in TCG (12 weeks), where the newly formed bone percentage in ROI2 slightly decreased 12 weeks after implantation (Table 1). The BSE-SEM micrographs are presented in (Figure 3), revealing the differences in both bone in-growth and bone on-growth as a function of group. In Figure 4, the new in-growth bone formation 4 and 12 weeks after implantation is shown.

### 2.4. Qualitative Evaluation in Cross-Section View

BSE-SEM high-resolution micrographs were first analyzed in real size in order to obtain an overall visualization. Afterwards, the same images were observed at increased magnifications by three independent researchers.

#### 2.4.1. Sample Groups 4 Weeks after Implantation

##### CG Group at 4 Weeks

In this group, a great difference among the samples was observed. An almost continuous thin line of newly formed bone tissue was observed in the cortical outer area for the vast majority of the samples. This bone layer originated in the preexisting cortical bone, and was continued by a limited number of trabeculae that penetrated the scaffold’s outer area (ROI1).

**Figure 5:**
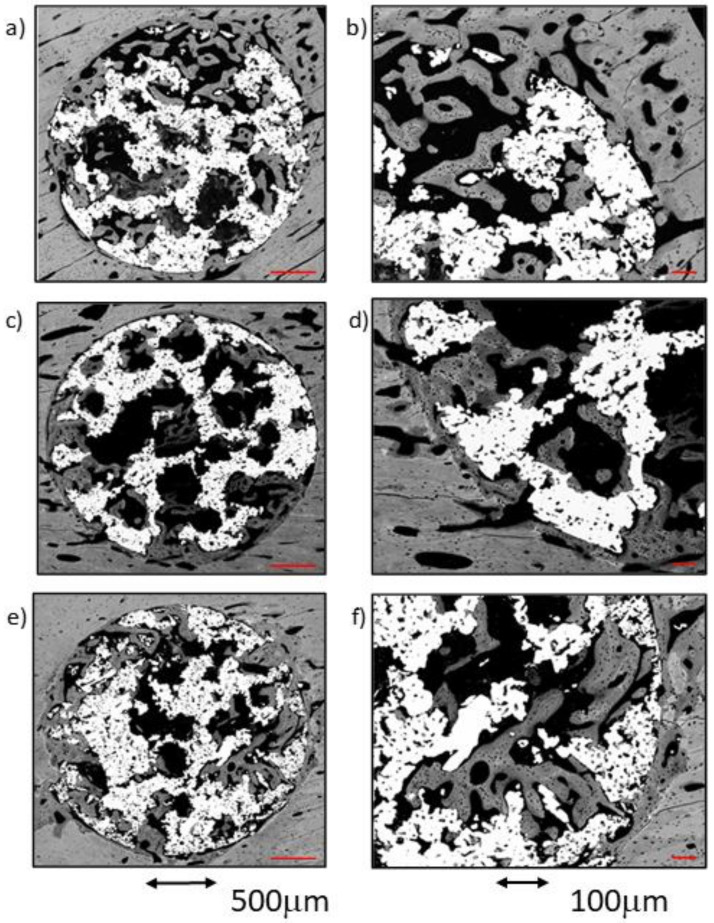
BSE-SEM micrographs 4 weeks after implantation in transversal section view at different magnifications: (**a**,**b**) CG; (**c**,**d**) TCG; (**e**,**f**) PAG.

The cortical surrounding the implant showed a notable increase in the number and size of the vascular channels, which appeared to be continuous with the ones surrounded by the trabeculae invading the scaffold’s outer pores. The trabeculae were constituted of an inner core of woven bone surrounded by a reduced number of layers of deposited lamellar bone, and were denser in ROI1 than in ROI2. Inside the implant, only a small volume of newly formed bone tissue on the surfaces of the pores at the central core of the implant (ROI3) was visible (Figure 5a,b).

##### TCG Group at 4 Weeks

As a rule, a notable amount of newly formed bone was observed in both the external area and inside the implant, coupled with an adequate bone-to-implant contact (BIC). The cortical surrounding the implant showed evidence of lamellar bone tissue remodeling, continuous with the bone tissues filling the scaffold’s peripheral pores (ROI1). The inner-core regions (ROI2 and 3) revealed the osseoconductive capacity of the newly formed bone that covered the outer surfaces of the scaffold’s deeper pores (Figure 5c,d). The osseous bone tissue that advances towards the center-core of the implant is woven bone; it prevailed in the outer area of the samples and established intimate contact with the titanium scaffold inside the porous structure. The lamellar bone remodeling of the scaffold’s peripheral pores (ROI1) was evident by the differences in the shape and orientation of the cell lacunae, and in the darker color of the extracellular matrix of the lamellar bone deposited around the vascular channels. The spare remnants of woven bone, lighter in color and with larger cell lacunae, were visible deep within the mass of new bone in the pores. Moreover, some images indicative of osteonal remodeling (Figure 5d), thus proving the maturity of its lamellar components.

##### PAG Group at 4 Weeks

A notable amount of newly formed bone tissue was generally observed in both the outer and inner areas of the implants in the PAG samples (Figure 5e,f). The thin, sparse trabeculae visible in the inner area of the scaffolds (ROI3) mainly constituted of woven bone, and had limited contact with the inner micropores of the scaffolds. However, the total amount of newly formed tissue, measured using the BIC values, appeared to be higher when compared to the other groups. The tissues observed in the outer area of the implants were continuous with the cortical osseous structures; in some samples a thin white line, the cementing line, signaled the initial osteoclastic resorption preceding bone apposition. The dense, well-connected, newly formed trabeculae, mainly constituted of lamellar bone deposited around what appeared to be vascular spaces, originated in the cortical surrounding the implant. Some osteonal images of the lamellar bone located between the preexisting cortical and the outer scaffold area (ROI1) are presented.

#### 2.4.2. Sample Groups 12 Weeks after Implantation

##### CG Group at 12 Weeks

The numerical analysis indicated great variability between samples. In general, the percentage of newly formed bone tissue at 12 weeks after implantation showed a slight increase compared to that for the same group at 4 weeks. Adequate BIC values were measured at this time point for comparison with the group at 4 weeks. Nevertheless, there was a limited amount of bone tissue inside the central core (ROI3) and the inner ring area (ROI2) of the scaffolds. Only in two samples of this group was bone tissue observed to reach the center of the implant.

The newly formed bone tissue was mostly found in ROI1, in the form of thick trabeculae, in which scarce remnants of woven bone were surrounded by lamellar bone. The lamellae were mostly deposited in parallel lines within the scaffold’s pores, while some images indicative of initial osteonal remodeling.

All CG samples showed intimate and adequate bone-to-metal contact, evidenced by the continuity of the remodeling layer around the implant, constituted of lamellar bone. The scaffold’s pores were filled up to 60%, mostly found in ROI1 and ROI2 areas, the inner core of the scaffolds being singularly devoid of bone apposition.

The longer implantation time for the CG group resulted in a greater presence of mature bone tissue, along with a higher amount of intimate bone-to-metal contact and higher micropore filling ratios.

##### TCG Group at 12 Weeks

The results of the analysis reflected great variability between samples in terms of the amount of newly formed bone tissue.

One of the samples was found to be almost completely filled with bone tissue, while the other two samples exhibited relatively little bone tissue inside the implant pores. Even so, all the implants showed newly formed bone tissue inside ROI3 in higher amounts than at 4 weeks after implantation.

**Figure 6:**
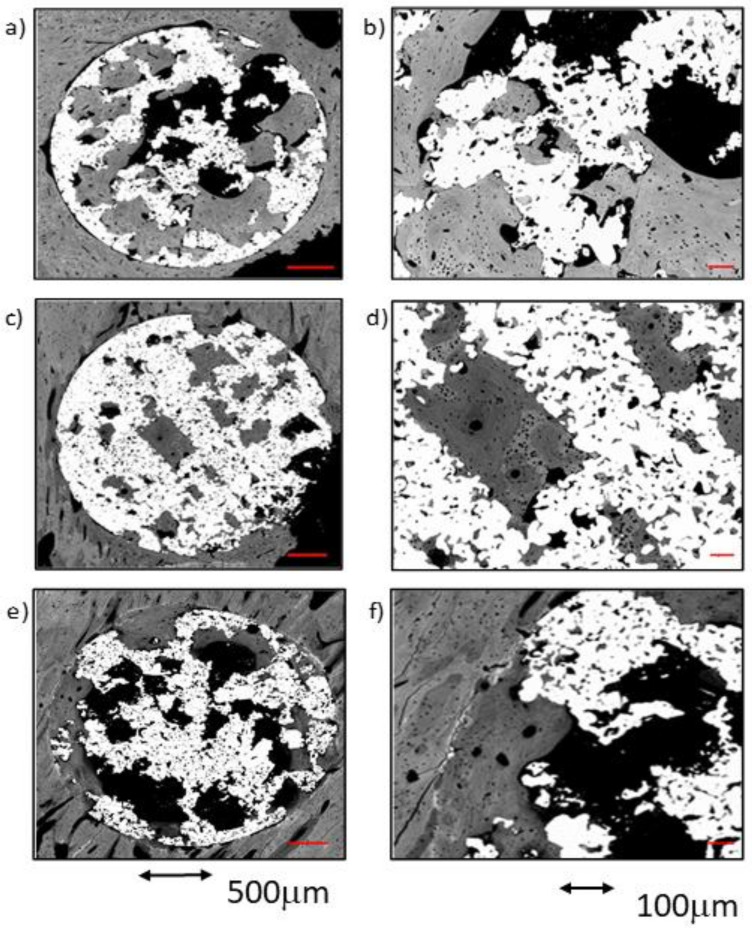
BSE-SEM micrographs 12 weeks after implantation in transversal section view at different magnifications: (**a**,**b**) CG; (**c**,**d**) TCG; (**e**,**f**) PAG.

The newly formed tissue showed an advanced status of maturity in both outer and inner areas of the implants. Very few remnants of woven bone were seen, while lamellar tissue was predominant in the majority of the dense trabecular bone structures that filled both the outer and the inner pores of the scaffolds (Figure 6c,d). The presence of osteons in the trabeculae proved the occurrence of active remodeling. Even in the center of the implants (ROI3), trabeculae were mostly constituted of lamellar tissue. Intimate contact of the newly formed bone tissues within the titanium was observed in most of the samples, inside the titanium macro- and micropores. In addition, the newly formed bone tissue appeared to spread towards the center of the scaffold by taking advantage of the titanium porosity. The increased implantation time of the TCG group resulted in a greater amount of bone tissue with a higher degree of maturity.

The comparison between the TCG and CG groups at 12 weeks reflected a slight superiority of the former in terms of tissue maturity, which showed less woven bone tissue in both the outer and inner areas of the implants. Furthermore, the TCG group showed intimate bone-to-implant contact over more implant surface than the CG group.

##### PAG Group at 12 Weeks

Compared to the rest of the groups at 12 weeks, the amount of newly formed bone between samples was moderately homogeneous in the PAG group. Overall, in all samples, there was newly formed bone tissue reaching towards the center of the implant. The bone-to-implant contact around the entire perimeter of the scaffolds constituted of lamellar bone, with only scarce remnants of woven bone. Moreover, it should be noted that this new bone tissue was continuous with the lamellar bone, filling the pores of the implant outer area (ROI1) and in the middle area (ROI2). The newly formed tissue had a high level of maturity in all evaluated regions, with a general prevalence of lamellar tissue with some osteons and scare evidence of woven bone tissue. Even in the inner core of the implants (ROI3), most of the bone tissue present was of the lamellar type. The increasing implantation time in the PAG group resulted in improved bone-to metal-contact, even if poor partial contact was reported in some areas. Newly formed tissue seemed to avoid contacting the titanium macropore surfaces, and achieved low penetration rates into the titanium micropores, regardless of the implantation time.

### 2.5. Qualitative Evaluation in Longitudinal View

SEM images were firstly analyzed in real size and then observed at increased magnification by three independent researchers.

#### 2.5.1. Sample Groups 4 Weeks after Implantation

##### CG Group at 4 Weeks

**Figure 7:**
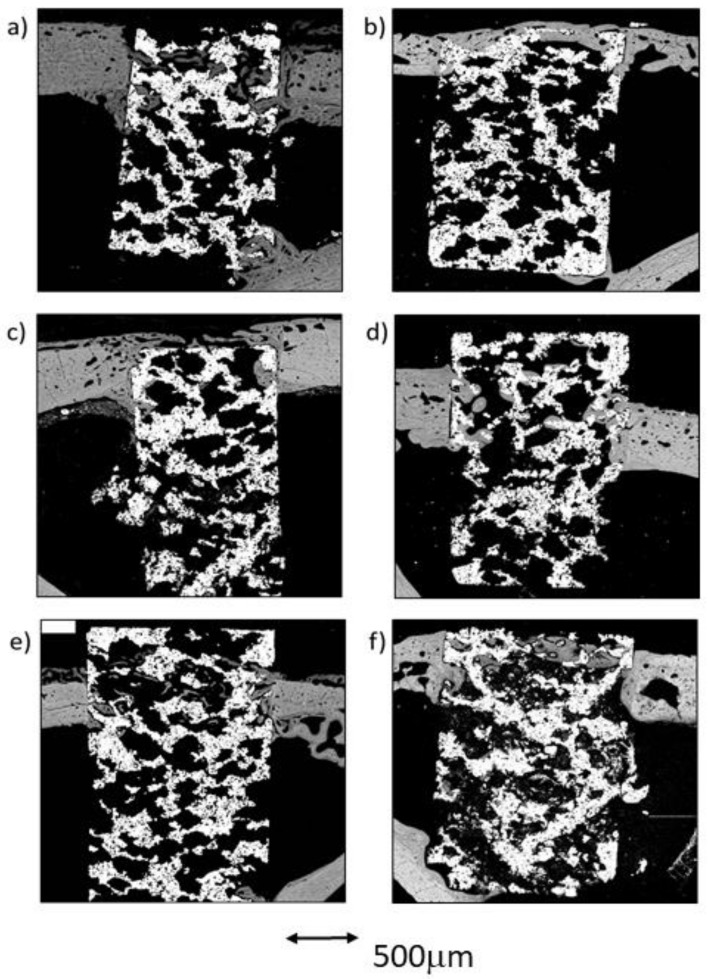
BSE-SEM results 4 weeks (left) and 12 weeks (right) after porous titanium implant insertion in longitudinal section view: (**a**,**b**) CG; (**c**,**d**) TCG; (**e**,**f**) PAG.

In the control group, it was discovered that the vast majority of the newly formed tissue was continuous with that found in the cortex. In two of the three samples, bone apposition was observed in connection with the endosteal cortical surface, but only some of the samples showed bone growth in connection with the exosteal cortical bone surface. The results presented in this section are very similar to those derived from the cross-sectional images of the same group, showing similar tissue maturity level, partial bone-to-implant contact, and acceptable micropore filling. Cementing lines were found separating the preexisting cortical from the remodeling bone. The structure of the newly formed trabeculae was similar to the that observed in the cross-sectional images; a thin layer of woven bone covered by lamellar bone. Some of these trabeculae connected the endosteous bone formation with the scaffold’s pores, both in the cortical sustaining the scaffold and in the contralateral cortical. Only a small amount of osseous tissue was observed in the central area of the implants (Figure 7a).

##### TCG Group at 4 Weeks

In the group of scaffolds constructed with thermochemical treatment, newly formed tissue was observed in the center of the implant in all samples. As in the control group, the newly formed tissue in the scaffold’s center was scarce. Likewise, most of the bone tissue found within the scaffold’s pores was continuous with the cortex, while a small amount of bone was also continuous with the endosteal remodeling area. Furthermore, two out of the three samples presented newly formed bone tissue coming from the periosteum. Figure 7c shows that the periosteal remodeling trabeculae completely covered the outer surface of the scaffold. These trabeculae were relatively thin and separated by ample space.

Lamellar tissue predominated the outer trabeculae alongside a small amount of woven bone tissue. At the center of the implant, lamellar tissue occupied most of the scaffold’s pores, surrounded by scarce remnants of clearer woven bone. Figure 7c also shows the intimate contact of the newly formed bone with the implant, through the substantial filling of the micropores situated nearer to the bone cortical.

##### PAG Group at 4 Weeks

In the three peptide-functionalized samples, newly formed tissue in the center of the scaffold was present. Thin trabeculae were visible within the scaffold, both in contact with the cortical endosteal and exosteal remodeling, and connected to some points on the scaffold’s pores. The trabeculae were mostly constituted of lamellar bone, surrounded by highly scarce remnants of woven bone, and separated by large spaces. The trabeculae were situated in line with the exosteal and all the endosteal remodeling areas, two in the supporting cortical and one in the contralateral cortical, and appeared to be more robust than those situated in the pores within the scaffold.

Lamellar tissue was also observed in the outer area, with a minor presence of woven bone tissue. Compared with the other two groups, the PAG group appeared to have the most mature newly formed tissue. Moreover, bone tissue growth could originate from the cortical bone, but also to a lesser degree from the endosteum or even the periosteum. As observed in the cross-sectional images, poor contact was made between the newly formed bone and the metal, as well as limited filling of the micropores, were observed.

#### 2.5.2. Sample Groups after 12 Weeks of Implantation

##### CG Group at 12 Weeks

The images obtained from the control samples after the 12-week postoperative period showed very heterogeneous results. In one of the samples, the newly formed bone tissue barely reached the center of the implant, whereas in the sample presented in Figure 7b, the remodeling process covered the center of the implant, while still in contact with both cortical borders. A dense mass of what appeared to be lamellar bone covered all the exosteal surface of the scaffold, while the scaffold’s pores seem to be filled with the tissues continuing the endosteal remodeling. Some very small woven bone remnants were seen within the lamellar bone.

##### TCG Group at 12 Weeks

The longitudinal in-growth analysis of the TCG samples showed bone growth as thick trabeculae in contact with the remodeling of the bone cortical, as well as of the endosteal and the periosteal cortical surfaces. A limited quantity of bone tissue was observed inside the implants; however, in all samples, it reached the inner area corresponding to ROI3. The bone was mostly lamellar tissue in both the outer and inner regions of the implants. In addition, many osteons were observed, indicating a high degree of remodeling in the surrounding osseous cortical. Again, the newly formed tissues were in direct contact with the metal surfaces, especially inside the implants. The newly formed tissue seemed to take advantage of the metal surfaces to spread towards the inner core of the scaffolds. In comparison with the CG group at 12 weeks, the TCG group’s newly formed tissue expressed higher maturity, greater bone-to-metal contact, and higher micropore filling rate values.

##### PAG Group at 12 Weeks

The longitudinal analysis showed high variability of results between samples. In general, a small amount of newly formed tissue was observed, but it connected the central core (ROI3) of the implants with the cortical remodeling in all samples. In two samples, this bone longitudinal in-growth came only from the cortex, and, in one of them (Figure 9f), a discrete amount of osseous growth coming from both the endosteum and the periosteum was also observed. The newly formed tissue was mostly lamellar tissue, and almost no areas of woven bone tissue were observed either outside nor inside the scaffolds. Again, a small amount of contact between the newly formed bone and the metal surfaces was observed, with only a partial filling of the micropores. The newly formed tissue was in direct contact with the bone cortical, but not with the metallic surfaces, thus filling the inner core of the scaffolds through the open spaces of the interconnected porosity.

### 2.6. Statistical Analysis

No statistically significant differences were found between groups. All the obtained values had a *p*-value > 0.05. No significant differences were found at the outer area level, for BIC, total newly formed bone, or when comparing the different ROIs between them. The obtained results are showed in Table 2.

## 3. Discussion

Titanium and its alloys are widely used for various implants in the orthopedic and dental fields due to their high biocompatibility and suitable mechanical properties. However, the elastic modulus of titanium is higher than that of living bone; therefore, it may induce bone resorption and stress shielding, following the bone mineralization guidelines that depend on load distribution, described in Wolff’s Law, and according to mechanical stimuli. For this reason, titanium implants with internal and interconnected pores are developed in order to decrease their elastic moduli to within the cancellous bone range (around 0.55 GPa), such as the implants used in this study [93].

The porous titanium implants used in this study were manufactured via sintering, using NaCl particles as a space holder agent to generate internal and interconnected porosity. These particles were easily removed by washing with distilled water, as they are water soluble, providing a cheap manufacturing method relative to others, such as selective laser fusion [94,95].

According to previous studies, some researchers have observed that the proper porosity for porous titanium implants should range between 25 and 66% [96,97] in order to stimulate osteointegration. Takemoto et al. in 2005 suggested that porous Ti with 40% porosity could also be a valid alternative for clinical use [98]. In the present study, the interconnected porous implants presented a total porosity of 53%, enhancing the biological response due to the high porosity benefits, including: facilitating the transport of body fluids and nutrients; aiding cell propagation inside the implant; promoting bone tissue proliferation and maturation in the scaffold structure. However, the balance between the porosity and mechanical properties is key and must be maintained [99].

The porosity and interconnectivity features are important since they can modify the mechanical properties and the biological performance, as well as the stability and fixation of the implants. The internal interconnection provides tunnels inside the implant that bone cells and extracellular matrix can colonize and, subsequently, enable neovascularization, thereby enhancing osseointegration and osteoconduction processes [37]. Nonetheless, nowadays there is still some controversy regarding the optimal pore size; when reviewing the published literature, most articles mention macropores between 100 and 400 µm as being the optimal size to enhance osteointegration [20,100,101]. A reasonable argument to justify these distinct values may be that most of these studies only focused on average pore size and did not consider pore interconnectivity, which is the communication channel between the macropores and a critical factor; for example, macropores with suitable diameters but poor interconnectivity will not permit new bone formation and neovascularization inside the implant. Channels directed towards neighboring macropores contribute to maintaining newly formed bone tissue in-growth under optimal conditions [98,102,103,104]. In the present study, the porous titanium implants showed an average pore diameter between 300 and 600 µm, with an average interconnectivity diameter of 210 µm, which allows the implant to be colonized by cells and newly formed bone tissue towards the inner scaffold space.

Although in the vast majority of similar studies, samples are analyzed using a single section plane, in the present study, the samples were evaluated considering two different planes; most of the samples were processed following cross-sections planes, while a smaller group was processed following longitudinal sections along the major axis of the implants. The main objective of this double-plane approach was to obtain the maximum information from the processed implants in order to acquire relevant data on the bone response that takes place inside the implants. Using this double-plane approach, a better quantitative assessment of osseointegration and osseoconduction was carried out because the penetration of the newly formed tissue in the innermost areas of the implant was thoroughly analyzed. On the other hand, the longitudinally observed samples allowed a better qualitative evaluation on the topographic origin of the bone tissue (periosteum, cortical, endosteum) and its pattern of propagation into the implant, which was evaluated in detail.

Based on our high-resolution SEM images, an innovative method that allowed automated image evaluation for interconnected porous titanium implants following different ROIs was developed and successfully implemented in this study. In this regard, different implant areas were digitally and automatically defined and subsequently analyzed using ImageJ to calculate the quantity of bone tissue in each ROI and in the bone–implant interface. To date, there is no scientific paper available with a detailed protocol outlining how to systematically evaluate osseointegration in and on porous titanium implants. The only authors who briefly described their methodology for analyzing porous samples were Takemoto et al. in 2005 [98], who cited the use of Photoshop and ImageJ to determine the bone in-growth area rate and the bone affinity index. Based on the information published by Takemoto et al. [98], we designed an automated method that created different regions of interest, using three ROIs inside the implant. Likewise, we created the concept of the outer area to define the newly formed bone tissue on the external implant perimeter. We also evaluated the BIC (bone-to-implant contact) to assess implant fixation by the peripheral, newly formed bone in a similar manner to the method defined by Manresa et al. [105]. Using this method, it was observed that all samples from all the groups exhibited new bone tissue formation inside the implant. In general, the peptide-coated group showed better qualitative and quantitative results compared with the thermochemically treated and control groups in terms of bone in-growth. However, the thermochemically treated samples exhibited notably higher adhesion between the newly formed bone and the metallic surface.

In view of our results, the PAG group would have reached slightly higher in-growth values than the TCG group. However, the TCG group had BIC values higher than the PAG group for the internal areas of the implant, which would ensure greater fixation of the implant, as well as a greater and more efficient mechanical bone-to-implant transduction. The greater contact between the newly formed tissue and the internal and external surfaces of the TCG implant would ensure greater transmission of mechanical loads to the bone, generating lower levels of stress shielding to the bone and, consequently, lower values of long-term bone resorption.

Both types of treatments efficiently stimulated the growth of newly formed bone tissue, both on the external surface of the scaffold and towards its inner porous structural core, even causing tissue in-growth from the cortical, periosteal, and endosteal bone tissue, in some cases.

It has been reported in many scientific papers, both in vitro and in vivo studies, that porous titanium alone, without any treatment, has osseoconductive and osseointegrative properties [20,93,94,106]. In this study, 4 weeks after implantation, the presence of woven bone tissue was observed, together with lamellar bone tissue in some areas of the samples from the control group both within the macro- and micropores. After 12 weeks, the results showed more mature newly formed bone tissue growing towards the inner areas of the scaffolds, with a predominance of lamellar bone tissue.

Even so, stronger results were obtained with the thermochemically treated group compared with the control group, both in terms of in-growth and newly formed bone in the outer area. There are many scientific articles that have used thermochemical treatment on porous titanium surfaces because it is an inexpensive and simple process. Briefly, when the implant is immersed in NaOH solution, it spreads over all the irregular implant structure, achieving a homogeneous bioactive surface, both on the outer part of the implant and in the innermost pores. Furthermore, the thermochemical treatment does not reduce the pore space available for bone tissue growth, since it only produces a thin coating of 500 nm needle-like sodium titanate structures on the surface of the pores, reducing them by a maximum of 1 µm [107] in diameter. The thermochemical treatment induces the formation of a dense and uniform apatite layer in contact with body fluids via ion exchange, which is similar to the bone mineralization phase. Then, thermochemically treated implants are attached to living bone through this apatite layer, providing not only the strong bonding of the apatite layer to bone tissue, but also a uniform gradient of stress transfer from bone to implant [108]. Numerous in vitro studies carried out during the last few decades have demonstrated the formation of this apatite layer on the implant surfaces [108,109,110]. In addition, several in vivo studies proving the notable effectiveness of thermochemical treatments to improve osseointegration and osseoconduction, have been reported [66,98,111]. In the present study, TCG implants exhibited increases in the growth of newly formed bone tissue, more mature bone tissue, and higher intimate contact and BIC values between bone and implant when compared with the CG samples. These results can be attributed to the homogeneous distribution of the treatment, covering all the sample’s macro- and micropores.

Very few scientific papers have been published that measure on-growth and in-growth bone formation and the intimate contact of the bone tissue with the metallic surfaces of the macro- and micropores. This fact has made it difficult to compare our results with other author’s reports.

The peptide-coating group showed the best results in terms of newly formed bone tissue quantity and maturity. The aim of peptide bioactivation is to immobilize certain peptide sequences on the implant surface to induce a specific cellular response, that is, to control the tissue–implant interface through the organic components of bone. This is accomplished through a group of cellular receptors called integrins that are involved in cellular adhesion by extracellular matrix proteins. Integrins interact with short amino acid sequences, in particular the Arg-Gly-Asp (RGD) sequence, which has been identified as a cellular adhesion mediator for plasma and extracellular matrix proteins [112]. Of the 24 known integrins, eight subtypes recognize and bind to the RGD sequence. Three of these eight subtypes are present in osteoblasts, so these sequences are used to promote the adhesion of osteoblasts to implants and thus improve osseointegration [112].

Therefore, peptides attached to implant surfaces have been demonstrated to improve cellular interaction with biomaterials [113]. The first studies dedicated to studying the effect of peptides attached to surfaces to bind osteoblasts began in the mid-1990s, and the first in vivo study with peptides was carried out in 1999 by Fernández et al. [6]. Nevertheless, there are very few published studies that assess the osseoconduction and osseointegration of porous titanium implants using peptides.

After an intensive literature search, no similar study was suitable for a proper comparison with our obtained data. However, this provides an innovative value to the present study, as it reports the first results of an in vivo study with porous titanium samples functionalized with a linear RGD peptide 12 weeks after implantation. In this study, the excellent performance of bioactive peptide treatments for increasing cell adhesion and proliferation, as well as for bone regeneration after a long-term postoperative period (12 weeks), is confirmed. Unfortunately, no reviewed study provides details on newly formed bone quantity, in-growth depth, or tissue quality for different time groups, as we did in this study. No reference, according to the findings observed in this study, related to the intimate adherence of the newly formed bone tissue to the internal macro- and micropores of the scaffolds was found either.

As a conclusion, both quantitative and qualitative analyses attained the best results for the samples biofunctionalized with the RGD peptide, showing the highest rates of newly formed bone tissue maturation and bone in-growth. However, a diminished intimate bone-to-implant contact inside the samples was also observed. This observation suggests some degree of peptide degradation or incomplete functionalization in the inner parts of the implant. On the other hand, the peptides may have greater availability on the outer surface. Thus, superficially, biologically active peptides stimulate bone tissue in order to grow and penetrate macro- and micropores; however, on a deeper level, these peptides fail to support intimate adherence of bone to the metallic surface. This would justify the good BIC values in the outer area of the implant, the high maturity and amount of newly formed bone tissue at the periphery of the samples, as well as the limited contact of the newly formed bone to the inside the implant.

In general, peptides are susceptible to enzymatic degradation by proteases, especially linear peptides, as they are more unstable in nature. A soluble linear peptide is degraded very quickly; however, when anchored to a surface, a steric hindrance is imposed on the enzymes, and therefore degradation is slower. A priori, it could be expected that the peptide on the outer area would degrade more rapidly than the peptide on the inner area, since it is more exposed to the medium. In this case, however, there may be more rapid cellular adhesion on the outer area, which is in direct contact with the bone tissue. This interaction is favored by the presence of tailor-made spacing units in the peptide sequence, which ensure its accessibility to the cells. It must also be assumed that the amount of RGD peptides is not the same within the entire implant, since silanization (the method used to bind the peptide to the metal) will be more efficient on the outer area than the inner.

If the two bioactivation mechanisms are compared, it can be seen that the two seem to stimulate osseointegration to a certain extent, although it could not be demonstrated with statistically significant differences. The mechanisms of action of an inorganic treatment, such as thermochemical treatment, compared to an organic one, such as peptide coating, are very different. The inorganic treatment promotes the nucleation of apatite crystals that are translated into "direct" bone formation, that is, the mineral part of the bone precipitates directly onto the implant. This is associated with bone growth from the implant outwards. In the case of peptides, RGD stimulates the integrins that promote adhesion, mechanotransduction, and finally differentiation and mineralization. Therefore, one could consider it as an indirect mechanism.

Regarding our review of the literature, we did not find any article describing the origin of the newly formed tissue, that is, if it grows from the cortical, the periosteum, or the endosteum. We believe that this is an important aspect since it can indicate the fixation success of the implant. If we only have bone tissue growth towards the interior of the implant, coming from the periosteum and/or the endosteum, we can deduce that the implant will not fix in the same way as it would with growth coming from the cortical. It would then be necessary to apply a large periosteal and endosteal bridge to stabilize the implant, and these bridges can strongly interfere with the clinical objective of the implant.

## 4. Materials and Methods

### 4.1. Implanted Materials

The samples used in this study consisted of porous titanium cylindrical implants 6 mm long and 3.5 mm in diameter, with open and interconnected porosity.

The scaffolds were produced using the pulvi-metallurgy (PM) technique by mixing grade 2 pure titanium (CP) particles, with an average mean grain size of approximately 80 µm, with NaCl particles ranging from 300 to 600 µm in diameter as a space holder, at a 65% volume ratio. The manufacturing process has been previously described [26].

Three different implant groups were generated according to its surface bioactivation method:

− Bioactivated porous titanium samples by thermochemical treatment (TCG).
− Bioactivated porous titanium samples by peptide adsorption (PAG).
− Porous titanium samples with no treatment used as control (CG).

### 4.2. Surface Bioactivation

Two groups of samples were subjected to surface bioactivation by two different methods: one group by thermochemical treatment (TCG) and the other by peptide adsorption (PAG). The thermochemical procedure was performed by immersing the implant in 5M NaOH at 60 °C for 24 h, followed by drying at 60 °C for 24 h, and finally the implant was heat treated at 600 °C for 1 h. This treatment enhances calcium phosphate precipitation over the implant surface in contact with body fluid.

The peptide adsorption procedure was achieved via covalent grafting of an RGD peptide onto the scaffold surface by means of silanization, as previously described [26]. In more detail, the peptide comprised of the cell-binding sequence Gly-Arg-Gly-Asp-Ser (GRGDS), three units of 6-aminohexanoic acid (Ahx) as a spacer [87], and 3-mercaptopropionic acid (MPA), which provides a thiol group as an anchoring moiety to react with the silane layer.

### 4.3. In Vivo Experimentation

All animal procedures in this study were performed in compliance with the Guide for Care and Use of Laboratory Animals [114] and the European Community Guidelines for the protection of animals used for scientific purposes [115], and under the permission of the National Committee on Human and Animal Research (ref# UAB-CEAAH 2016).

The surgical procedure was performed under standard sterile conditions. Implanted materials were sterilized with gamma radiation at 7 KGy before surgery. The in vivo study was carried out in 18 female adult New Zealand White (NZW) rabbits (Charles River, France), with an average body weight ranging from 4.0 to 6.0 kg.

The experimental animals were randomly divided into two groups: Group A (4 weeks of postoperative period) and Group B (12 weeks of postoperative period), with 9 rabbits for each group. The three implant groups (TCG, PAG, CG) were randomly distributed by placing one sample per tibia.

### 4.4. Surgical Technique

The experimental animals were in optimal physical condition, and acclimatized individually for 2 weeks prior to surgery. Health status was determined by physical and orthopedic examination, radiographic examination of the rear limbs, and the results of hematologic and serum biochemical profiles.

For surgical procedure, the animals were preanesthetized using buprenorphine (0.03 mg/kg s.c.), midazolam (0.50 mg/kg s.c.) and medetomidine (0.05 mg/kg s.c.). Anesthesia was induced with propofol (2.5 mg/kg s.c.) and maintained via inhalation of isoflurane (2%) through an oxygen mask.

**Figure 8:**
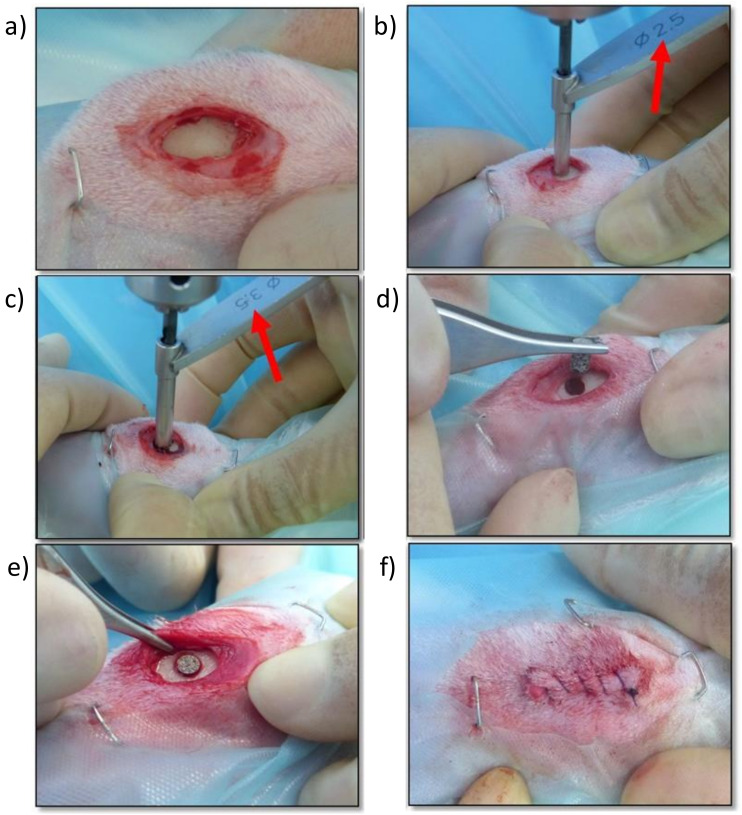
Screen photographic sequence of the surgical method: (**a**) Medial approach to the proximal aspect of the right tibia, (**b**) Creating a monocortical bone defect with a 2.5 mm drill bit, (**c**) Enlarging the monocortical bone defect with a 3.5 mm drill bit, (**d**) Placing the titanium implant in the monocortical bone defect, (**e**) Checking the adequate placement and fixation of the titanium implant, (**f**) Surgical wound sutured with a continuous pattern.

For the surgical implantation (Figure 8), with the animals in dorsal recumbency, both tibial regions were clipped and subsequently scrubbed with chlorhexidine gluconate solution (4%) as an aseptic preparation of the surgical field. Afterwards, the medial aspect of the proximal tibia was exposed through a limited skin and subcutaneous incision. Using drill bits of increasing size and under copious irrigation with physiological saline, a final monocortical bone defect of 3 mm in diameter was generated on a craniocaudal midpoint of the medial aspect of the proximal tibia. Cylindrical porous titanium implants were placed inside these defects using “press-fit” mode until flushed with the cortical surface. The subcutaneous tissue and the skin were sutured in layers using a standard suture pattern with synthetic resorbable sutures (Vycril 3/0, Ethicon, Johnson & Johnson, New Brunswick, NJ, USA). Immediately after surgery, and with the animals still under general anesthesia, tibial mediolateral and craniocaudal radiographs were taken to confirm the correct location of the implants.

### 4.5. Euthanasia

The animals were euthanized at the scheduled survival times with an overdose of sodium pentobarbitone (200 mg/kg i.v.) according to the legislation of the American Veterinary Medical Association. (AVMA). Pre-euthanasia sedation using midazolam (0.50 mg/kg s.c.) and medetomidine (0.05 mg/kg s.c) was conducted for animal welfare reasons.

Then, the tibiae bones were completely harvested and the peripheral soft tissue was removed. Finally, craniocaudal and mediolateral radiographs of the bone samples were performed.

### 4.6. Samples Preparation

Bone tibiae samples were individually identified and stored by immersion in neutral buffered 10% formalin solution for 3 weeks in order to assure proper bone fixation and tissue preservation. After the fixation time, samples were rinsed in running tap water for 15 min to eliminate any fixative agent residues. After rinsing, samples underwent a progressive dehydration process by immersion in increasing concentrations of ethanol in aqueous solutions (from 30 to 100% *v*/*v*) with constant stirring at 50 rpm. Once dehydrated, samples were immersed in ethanol solutions with increasing concentrations of methyl-methacrylate resin Technovit 7200 (Kulzer-Heraeus, Hanau, Germany) (from 30 to 100%) with constant stirring at 50 rpm. Finally, samples were kept under vacuum conditions for 24 h to ensure resin penetration into the tissues, and subsequently embedded in a 100% resin solution by photo-polymerization using a light control unit Histolux (Kulzer-Heraeus, Hanau, Germany), following a 24 h process of visible and UV light exposure.

A total of 36 samples were obtained and were cut in 2 halves to expose the metallic scaffold: 15 implants were cut perpendicularly to the longitudinal axis of the scaffold (transversal mode) and 3 implants were cut following its longitudinal axis (longitudinal mode). Exposed metallic surfaces were polished with SiC abrasive papers (800, 1200, 4000 index mesh) using a Exakt-400 CS grinding machine (Exakt, Norderstedt, Germany) to obtain smooth and scratch-less surfaces for SEM observation. For each postoperative period group, a total of 15 implants were used to obtain samples in scaffold cross-section mode, and 3 samples to obtain samples cut in scaffold longitudinal axis.

### 4.7. Obtaining Images of the Samples by SEM

The polished samples were carbon-coated by sputtering and then observed via scanning electron microscopy (SEM) using a Neon40 Crossbeam^TM^ FIB-SEM (Carl Zeiss, Dresden, Germany) with backscattered electron (BSE) detector. Observation conditions were at a voltage of 15 kV with a working distance of about 8 mm to achieve a resolution up to 1.1 nm in BSE-SEM mode.

The SEM observation was performed by carrying out a sequential scan of the surface, acquiring high magnification pictures, and then merged using SMART STITCH Software (Carl Zeiss, Dresden, Germany) and ImageJ 1.46 Software (NIH, Fredrick, MD, USA). The obtained images were individually processed using ImageJ (Adobe Systems, Dublin, Ireland) in order to ensure correct histomorphometry evaluation.

### 4.8. SEM Quantitative Evaluation Method

Stitched images obtained by SEM were calibrated and analyzed by using ImageJ software. All images were identically acquired (see SEM/FIB settings) and post-processed. Every image was thresholded into metallic implant area, void porous space, and newly formed bone tissue inside the initial defect created during the surgery procedure. Thus, thresholded binary images derived from the grey-scaled original image were created for each sample.

**Figure 9:**
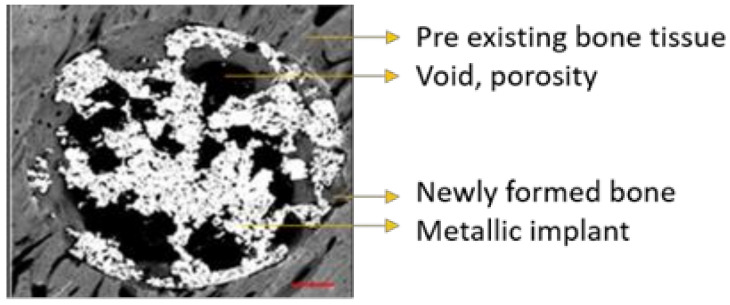
Stitched grayscale image obtained via SEM analysis detailing the different materials identified.

ImageJ was used to quantify the area (percentage) occupied by every thresholded tissue and/or material present in each sample from binary data, as follows: metallic implant area (white), void porous space inside the defect (black), and the newly formed bone tissue (grey scale) present in the free space available for bone growth, as can be seen in Figure 9. The latter was crucial in order to evaluate the amount of bone tissue generated within the internal and interconnected porosity, so the more porosity the more space available for the bone to grow.

Outermost newly formed bone tissue can be differentiated from pre-existing host bone tissue due to differences in grayscale values, microarchitecture (growth band display) and microstructure (maturity and porosity) of the tissues. The threshold applied to every tissue/area in every image was individually selected for each sample and performed by the same user. All samples were previously coded to ensure blind analysis.

The bone in-growth was characterized by the newly formed bone and was quantified in 3 different regions of interest (ROI) inside the implants: outer ROI (ROI1), middle ROI (ROI2), and center ROI (ROI3), where each ROI radius was a third of the implant radius (Figure 2). ROI analysis was performed taking into account the available space existent within the interconnected porosity to fill with newly formed bone. All newly formed bone inside the whole implant was quantified and referred to as “total in-growth”.

**Figure 10:**
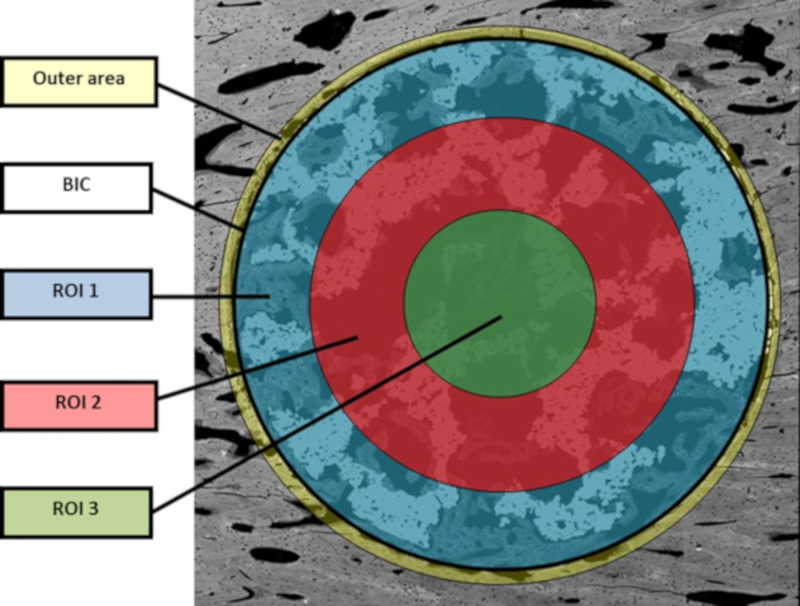
Graphical scheme for assessment of BIC and ROI values.

The bone on-growth assessment was performed by “outer” bone and BIC (bone-to-implant contact) parameters. The outer bone was identified as the newly formed bone around the implant, from the external implant perimeter up to 100 µm radially towards the outside. The bone tissue loss, due to thermal necrosis caused by surgical milling and by the vascular ischemia induced during the implantation, was taken into account. BIC was evaluated as the direct contact between mineralized, newly formed bone and the external implant surface. A graphical scheme is shown in Figure 10.

All samples were analyzed with the same methodology. ROIs were automatically drawn to be equivalent in whole samples. All samples were evaluated by 3 different independent researchers, first in real size and then in detail at different magnifications.

### 4.9. Qualitative Evaluation for Transversal Images

The following parameters were analyzed:

- Quality and maturity of external newly formed bone.
- Quality and maturity of external newly formed bone in direct contact with external implant surface (bone-to-implant contact, BIC).
- Quality and maturity of external newly formed bone inside the interconnected scaffold porosity.
- Penetration of newly formed bone inside the scaffold.
- Newly formed bone contact within the metallic scaffold surface.
- Bone filling inside the scaffold microcavities and micropores.
- Assessment of the bone progression towards the scaffold center.
- Presence of necrotic or soft tissue.

### 4.10. Qualitative Evaluation for Longitudinal Images

Three samples of each postoperative period group (4 and 12 weeks) were used to obtain longitudinal images and perform a qualitative analysis. The newly formed bone tissue and its origin (periosteum, cortical, and/or endosteum) were analyzed in the three experimental groups (TCG, PAG, CG).

### 4.11. Statistical Analysis

Histomorphometrical results were set out as mean ± standard error of the mean. All numerical values followed a normal distribution, as Anderson–Darling and Kolmogorov–Smirnov tests confirmed.

Statistical analysis was performed using a one-way ANOVA followed by Tukey post hoc tests using GraphPad Prism software (ANOVA, La Jolla, CA, USA). Statistically significant differences were considered as *p* < 0.05.

## 5. Conclusions

Albeit after both 4 and 12 weeks of implantation, no statistically significant differences were observed between the untreated scaffolds and the bioactivation treatments, quantitative analysis revealed higher values for the biotreated surfaces when comparing ROIs, especially for the outer area, as well as the inner volume filling of the scaffolds.

However, when quantitatively comparing the two bioactivation methods, the peptide treatment strategy seems to be the best option to enhance and accelerate bone tissue growth over the implant surface according to the BIC values achieved, while the thermochemical treatment strategy yielded better filling values in the inner core areas of the scaffolds. These results suggest that both strategies contribute towards enhancing porous metallic implant stability and osteointegration, and a combination of both strategies might be worth pursuing.

From a qualitative point of view, the bioactivation of titanium implants through the innovative peptide treatment used in this study generated an osseoregenerative response, which constituted of a greater amount of more mature, newly formed bone tissue, when compared with the responses obtained from the thermochemical and control groups. However, this greater and rapidly maturing new bone formation is not accompanied by a greater adhesion to the titanium pores within the inner core of the implant.

Concerning to the implantation time, the presence of woven bone tissue was observed 4 weeks after implantation, together with lamellar bone tissue in some areas of the samples from the control group both within macro- and micropores. After 12 weeks, the results showed more mature, newly formed bone tissue growing towards the inner areas of the scaffolds, with a predominant presence of lamellar bone tissue.

Furthermore, the whole innovative experimental design, including the protocol for the surgical insertion of implants, and the protocol for digital histomorphometrical assessment and tridimensional evaluation of the scaffolds, proved to be both efficient and effective. The sequence of quantitative results allows a comprehensive analysis of the advancement of the osseointegration process, and sustains the ulterior qualitative observation of the maturity and ultrastructural patterns of bone regeneration. This way, the reported protocol for the assessment of the evolution of osseoconduction and osseointegration of the implants can be considered as an evaluation standard for subsequent studies with similar objectives.

## Figures and Tables

**Table 1:** Overall SEM histomorphometric results table in transversal section.

Group		BIC	OUTER On-Growth	INNER In-Growth			
Temporal	Treatment	(%)	External (%)	ROI1 (%)	ROI2 (%)	ROI3 (%)	TOTAL (%)
4 Weeks	GC	49.24 ± 10.78	63.64 ± 7.06	28.18 ± 7.21	8.34 ± 2.58	6.33 ± 1.47	13.48 ± 3.47
	TCG	56.87 ± 3.70	64.45 ± 4.48	34.84 ± 3.85	13.96 ± 2.06	6.59 ± 0.62	18.05 ± 2.00
	PAG	58.07 ± 3.60	68.54 ± 4.40	39.55 ± 3.75	16.73 ± 5.34	8.78 ± 3.98	21.49 ± 3.67
12 Weeks	GC	59.05 ± 2.66	67.79 ± 2.77	43.47 ± 3.03	8.75 ± 2.85	10.61 ± 8.35	20.94 ± 4.48
	TCG	60.91 ± 5.93	71.60 ± 1.93	43.31 ± 5.58	15.14 ± 4.49	16.16 ± 5.62	24.87 ± 3.39
	PAG	68.93 ± 4.13	74.55 ± 4.71	46.03 ± 5.10	16.41 ± 5.39	11.53 ± 5.04	24.66 ± 4.11

**Table 2:** Overall comparative statistical table of results.

Area	Temporal Groups	Treatment Groups	Significance	*p*-Value
**OUTER AREA**	4 WEEKS	CG vs. TCG	NO	0.923
		CG vs. PAG	NO	0.563
		TCG vs. PAG	NO	0.533
	12 WEEKS	CG vs. TCG	NO	0.291
		CG vs. PAG	NO	0.251
		TCG vs. PAG	NO	0.578
**INNER AREA**	4 WEEKS	CG vs. TCG	NO	0.276
		CG vs. PAG	NO	0.131
		TCG vs. PAG	NO	0.434
	12 WEEKS	CG vs. TCG	NO	0.504
		CG vs. PAG	NO	0.558
		TCG vs. PAG	NO	0.969
**BIC**	4 WEEKS	CG vs. TCG	NO	0.977
		CG vs. PAG	NO	0.908
		TCG vs. PAG	NO	0.822
	12 WEEKS	CG vs. TCG	NO	0.782
		CG vs. PAG	NO	0.079
		TCG vs. PAG	NO	0.300

## Data Availability

The data that support the findings of this study are available from the corresponding author upon reasonable request.
